# Development and usability testing of mobile application on diet and oral health

**DOI:** 10.1371/journal.pone.0257035

**Published:** 2021-09-08

**Authors:** Noor Akmal Muhamat, Ruhaya Hasan, Norkhafizah Saddki, Muhammad Rafie Mohd Arshad, Mokhtarrudin Ahmad

**Affiliations:** 1 School of Dental Sciences, Universiti Sains Malaysia, Kubang Kerian, Kelantan, Malaysia; 2 School of Computer Sciences, Universiti Sains Malaysia, Pulau Pinang, Malaysia; 3 Faculty of Applied Communication, Multimedia University, Cyberjaya, Selangor, Malaysia; Griffith University, AUSTRALIA

## Abstract

In several nations, caries in pre-school children remain a significant oral health issue. In an outbreak period such as the Coronavirus disease 2019 (COVID-19), remote contact and education aimed at the prevention of oral diseases and the preservation of children’s oral health are more relevant than ever. Currently, the amount of published applications is far higher than the published scientific studies while the problems of usability remains vulnerable. The goal of this paper was to comprehensively document the phase of development and usability testing of a mobile application for diet and oral health, namely *Gigiku Sihat*, which was primarily intended to be used by parents and guardians of pre-school children. The mobile application was developed using the System Development Life Cycle principle. Apart from searching for the available oral health application on Android platform, the initial requirement gathering process consisted of situational analysis, concept generation, content development, and features and functional requirement determination. The mobile application design and implementation evolved at each phase before being finalised. *Gigiku Sihat* was successfully developed in the Bahasa Malaysia. Finalised *Gigiku Sihat* was installed on mobile devices to determine the usability using translated and validated System Usability Scale questionnaire namely *Skala Kebolehgunaan Aplikasi Mudah Alih* (SKAMA). The mean score usability with score of 68 and above was deemed to have good usability. This study found that *Gigiku Sihat* mean (SD) usability score was 77.0 (14.18). The results were promising as they showed that *Gigiku Sihat* had a good usability. Thus, the development of this mobile application focusing on diet and oral health served as a new source of oral health education and provided a necessary foundation in developing future improved mobile application development for parents in the prevention of early childhood caries.

## 1. Introduction

Given the decline in the prevalence of dental caries in children of Western countries, dental caries in pre-school children remain a significant concern in both emerging and industrialized countries [1]. The most recent national epidemiological survey in 2015 found that the caries prevalence of Malaysian pre-school children was 71.3% [2]. The national reports stipulated high caries level found in deciduous teeth as well as a high unmet treatments [3]. Efforts to prevent diseases are inherently preferable to dental rehabilitation from the standpoint of civil rights, health promotion and social justice [4]. Furthermore, the challenge of managing regular dental check-ups and follow-ups in an outbreak situation such as the Coronavirus disease 2019 (COVID-19) makes it important to concentrate on prevention through oral health education interventions with sufficient remote information.

A systematic review and meta-analysis by Stein et al. (2017) had found that traditional oral health education using collective actions such as lectures, slides, counselling, drawing, dietary guidance, albums, leaflets, games, theatre and other types of actions such as oral hygiene instruction, supervised tooth brushing and tooth brushing demonstration was effective in reducing plaque accumulation over a short period of time [5]. Although traditional oral health education has been shown to be effective in changing health behaviours [6, 7], traditional approaches can present a number of challenges, including logistical issues, the challenge of keeping participants actively engaged, and being labour intensive and expensive to scale for larger populations [8]. Because of their cheap cost, great reach, anonymity, flexibility, and scalability, digital environment components may provide answers to traditional problems [9].

Mobile health (mHealth) is defined by the World Health Organization’s Global Observatory for eHealth as public health and medical strategies assisted by mobile technology such as mobile phones, and it involves the exertion and usage of more complex functionalities and applications in additional to the simple mobile phone services [10]. A review of published literature concluded that smartphones would be the primary medium for mHealth applications [11]. In terms of effectiveness, a series of interventional studies on oral health-related mobile application targeted various research population, including autism spectrum patient [12], school teachers [13] and teenagers with fixed orthodontic appliances [14, 15]. Fewer research focused on mothers of children under the age of six [16] and pre-school children [17, 18]. There have also been studies leveraging popular messaging application such as Telegram [19] and Whatsapp [20] to deliver and receive feedback on oral health messages. Following that, different outcomes from the oral health-related mobile application research were investigated, such as improving dentist-patient communication [12], improving patient compliance [20], increasing knowledge through its educational content [13, 16] and improving oral hygiene and oral habit through its persuasive content [14, 15, 17–19].

However, the existing mHealth applications are deficient in ground-based theories and assessment, security and usability problems are still very much fragile and the amount of published software is far higher than published scientific literature [11]. In terms of content, Sharif and Alkadhimi assessed twenty oral hygiene-related and commercially available mobile application. It was found that although the mobile application scored highly for functionality, the information content quality scored lowest and suggested further improvement [21]. Issues on information with regards to their approval by official organisations and whether effectiveness or acceptability testing had been conducted were also raised [22, 23]. Khatoon et al. [24] also indicated that there was a need for peer-reviewed, validated and high quality content for the enormous amount of available smartphone applications for oral health education. In contrast, Fijacko et al. assessed seventeen mobile application that focused only for children and found that they were highly rated in quality appraisal. The mobile applications include gamification features and behavioural change technique in order to perform and maintain the children’s own oral hygiene [25].

It is generally accepted that nutrition and diet should be included for a holistic health program because they are common risk factors for oral diseases [26]. Literature reviews on the usage of applications to enhance nutrition-related behaviour and increase nutrition knowledge found that smartphone applications were likely to be a low-cost and valuable intervention [27, 28]. However, very few studies have examined the use of nutrition applications as supportive educational interventions beyond food record keeping [28]. Furthermore, culturally adapted research-tested mobile applications are needed and suitable for non-English speakers and for people with lower health literacy, thus requiring the behaviour theory within the application [27, 28]. In terms of effectiveness, a significant reduction in the mean intake of sugar-sweetened beverages was identified in the intervention using mobile application for school and pre-school children [29, 30]. Using the "Sugar Smart" software that enabled consumers to identify total sugar in various food items by checking the barcode of products, the total sugar intake had decreased by 6.2 g/day at peak campaign and the percentage of energy from total sugars significantly decreased immediately and 1 year post campaign [31].

Because oral health of the children is closely linked to the circumstances and lifestyle of the child’s caregivers, early intervention programs should include their parents [32, 33]. This research is required since little is reported about the state of the currently accessible oral health-related smartphone application in Malaysia, and there are no prior studies available on the overall development and usability of the current mobile application that focuses on both diet and oral health for the prevention of early childhood caries. This paper aimed to comprehensively document the development and usability determination process of mobile application, which specifically directed towards parents and guardians of pre-school children, on children’s diet and oral health.

## 2. Materials and methods

### 2.1 Development methodologies

The development and usability determination process of a mobile application on children’s diet and oral health, namely *Gigiku Sihat* which means “my healthy teeth” was carried out from December 2018 until August 2019. It was developed in Bahasa Malaysia on the Android platform. The Android platform was selected because the accessible survey data revealed that it was Malaysia’s biggest smartphone platform [34]. The method of developing a mobile application was adapted from the principle of the Software Development Life Cycle (SDLC) which consisted of five phases such as the requirement gathering, design, implementation, testing, and maintenance [35]. Studies have described the use of SDLC in mobile application development [36–38] and an integrative literature review has also found that SDLC was one of the main methods for developing mobile application in the area of health [39]. SDLC is essentially a whole process with structured and rational actions required to create software products [40]. This paper would only address the requirement gathering, design, implementation and testing phases of *Gigiku Sihat*.

#### 2.1.1 Requirement gathering

First and foremost, the accessible oral health mobile applications on Android platforms have been checked to decide the novelty of the idea and the supported features have been recorded. The search was conducted in December 2018. Keywords used included ‘oral’, ‘mouth’, ‘tooth’, ‘teeth’, ‘dental’, ‘dentistry’, ‘mulut’ and ‘gigi’. Eighteen mobile applications were selected based on the latest releases or updates on Google Play Store. They were downloaded, and the information were recorded. It was noted that majority of the mobile applications focused on common oral health and only three were developed in the local language, Bahasa Malaysia. None of the local mobile applications focused on children diet and oral health, and specifically targeted their parents or guardians. The most frequent features found showcased information, game or quiz or reward and data register or directory.

The team opted for user-centred approach for the mobile application design where the design activities and processes still mainly focused on users’ needs without assigning active role to the users. In addition, there were also members of the team who were parents of pre-school children and represented the users. We have adapted the Behaviour change theories, User-centered design, Social marketing (BUS) Framework suggested by Patel and Arya [41] to optimize mobile application efficacy during the requirement gathering process. In additional to content and function requirement, BUS framework steps were adapted for the requirement gathering phase which involved situational analysis and concept generation. Detailed steps were taken as follows:

*2*.*1*.*1*.*1 Situational analysis*. Situational analysis was conducted accordingly by performing literature review as a part of the study. Information was collected using the secondary (pre-existing) data collected from national surveys and previous studies. It was reported that dental caries was a significant oral health issue faced by pre-school children in Malaysia as more than 70% of them were already afflicted by the disease at this early age [2, 42]. While it was mainly preventable, it was also reported that children in Malaysia had high sugar intake, which exacerbated the condition as sugar was an important dietary element in the dental caries development [43].

Since its inception in 1984, an oral health program for pre-school children in Malaysia has provided oral health education to pre-school teachers and children [44]. Thus, the research team aimed to approach these pre-school children’s oral health issues from a particular perspective. The end users or target population of the *Gigiku Sihat* mobile application was parents of pre-school children. Overall, global research has shown that parents have a poor required knowledge level of pre-schooler’s oral health and hygiene to maintain the proper oral health and reduce the risk of early childhood caries [45]. Meta-analysis by Fedele et al. [46] showed that the intensity of the intervention impact improved by delivering mHealth intervention to the caregivers. Subsequently, once literature review was done, we began to engage with an interdisciplinary team including dental public health practitioners, nutritionist, information technology (IT) expert, communication expert, designer and mobile application developer to kickstart the development of the prototype mobile application of *Gigiku Sihat*.

*2*.*1*.*1*.*2 Concept generation*. Following the recognition of the oral health issue after a situational analysis, the concept was chosen. The constructs of behavioural modification theory were used to develop a prototype that satisfied the expectations of end-users. After a thorough consideration of the future research trial and the target demographic, the concept of oral health education was considered to be acceptable in order to increase diet and oral health knowledge, attitudes and practice among parents in the prevention of caries, and to enhance dietary and oral hygiene among their pre-school children. For the conceptual basis to endorse *Gigiku Sihat’*s content, we used a rational model and a health belief model [47, 48]. These models have been previously applied in several mHealth interventions studies [49–52].

The rational model, also known as the Knowledge, Attitudes, Practice Model (KAP), is focused on the idea that increasing a person’s knowledge can contribute to behavioural improvement [53]. Meanwhile, the health belief model discusses human views of the danger presented by the health issue (susceptibility, severity), the advantages of preventing the threat, and reasons that affect the decision to act (barriers, cues to action, and self-efficacy) [54]. Applying a health belief model to an oral health problem such as early childhood caries, parents must believe that the child is vulnerable to dental caries, that primary teeth are vital and dental caries are a significant danger to them, and that dental caries may be prevented, and must be prepared to restrict the child’s access to fermentable carbohydrates, and must assist the child in practicing good oral hygiene [55]. Potential change strategies incorporated in the *Gigiku Sihat* mobile application are shown in Table 1. While considering the target population, the concept was translated into content of the mobile application *Gigiku Sihat* which focused on diet and oral health among pre-school children.

**Table 1:** Potential change strategies in *Gigiku Sihat*.

Concept	Potential Change Strategies
**Perceived susceptibility**	Users will be initially presented with brief information on dental caries prevalence and sugar consumption in pre-school children in the “About” section.
	Tailored information for factors that cause dental caries and level of risks by food type for local users.
	Use of images to help the users develop an accurate perception of their children’s risk of dental caries.
**Perceived severity**	Specify the consequences of dental caries in the children.
**Perceived benefit**	Explain how, where and when to take effective and recommended action specific for their children age, and what the potential positive result will be included in the “prevention of dental caries” section.
**Perceived barriers**	Extensive evidence-based information to facilitate access to diet and oral health information and to correct misinformation.
**Cues to action**	Generate cues by sending local (push) notifications to the display of the device at programmed intervals to promote awareness and remind users on the children’s diet and oral health care.
**Self-efficacy**	Provide guidance in the performing action in mobile application content and “frequently asked questions” section.
	Demonstrate desired behaviours via images, illustrations and videos.

*2*.*1*.*1*.*3 Content development*. Generally, the elements of the content and the process involved are set out in Table 2. Firstly, information were gathered for the content creation in Bahasa Malaysia. Content writing was driven by the TIP TOP module [56], publications and guidelines from Ministry of Health Malaysia [57, 58] and written articles and books by oral health experts [59, 60]. The research team conducted a face-to-face strategy meeting and picked four main oral health messages: oral hygiene, healthy diet, fluoride use and dental visits. Content write-up was drafted by the researcher, revised by the oral health and nutrition expert, and subsequently finalised. The accepted contents of the main theme menu and the videos of *Gigiku Sihat* were seen in Table 3.

**Table 2:** Content elements and process involved.

Content element	Process involved
Write-up	i. Information gathering
	ii. Strategize
	iii. Draft, revision and finalise content write-up
Video	i. Pre-production
	• Draft, revision and finalise the storyboard and scripts
	• Preparation of location, material and equipment
	• Casting and consent
	ii. Filming
	• Recording of shots
	• Recording of voice over
	iii. Editing
	• Draft, revision and finalise video
Photographs and illustrations	i. Create own or procure third-party photographs and illustrations
Push notifications	Draft, revision and finalise notifications and reminders content

**Table 3:** Content of *Gigiku Sihat*.

Content	
**Topics**	1. Healthy diet
	2. Dental caries
	3. Causes of dental caries
	4. Effect of dental caries
	5. Prevention of dental caries
	6. Parents’ role
	7. Frequently asked questions
**Videos**	1. Oral hygiene method–birth to 6 months old
	2. Oral hygiene method– 6 months to 1 year old
	3. Oral hygiene method–brushing with toothpaste
	4. Lift the lip (oral health screening tool for parents to confidently check their children mouth for early sign of dental caries)

The lists and mock photographs and illustrations were already approved from the content write-up process. The photographs were then captured by the researcher team and illustrations were drawn using Adobe Illustrator and Adobe Photoshop. Photos on different types of toothbrush, toothpaste and intraoral as well as illustrations on tooth brushing technique and tooth structure were procured from a third-party. Lastly, for the content elements, push notifications and reminders content were drafted, revised and finalised by the researcher team. Overall, forty-five notification messages related to the diet and oral health topics and two reminder messages were approved.

*2*.*1*.*1*.*4 Features and functional requirement*. The researcher team also proposed the features, functional requirement and non-functional requirement for the mobile application. For *Gigiku Sihat*, the core functional requirements that were expected to be performed by the mobile application were to deliver local (push) notification, be able to save data locally and be able to record mobile application usage. If the participants did not use the mobile application, it would send personalized reminder through push notification every one week reminding them to use it. Push notification messages were able to deliver diet and oral health tips every two days. Meanwhile, the non-functional requirements included operation without internet coverage and a user-friendly mobile application. Once these requirements were conveyed to the developer team, they determined that the minimum Android software development kit (SDK) version required was SDK 16 Android 4.1 (Jelly Bean) and the budget to fulfil the requirements.

#### 2.1.2 Design

*Gigiku Sihat* was developed to achieve good user interface (UI) components. UI is a broad term for any system, either software-based or physical, that allows the user to link to the technology, which implies that it is what the user can communicate with and see. The aim of a UI design is to render contact with users as easy and effective as possible in terms of achieving users’ objectives (user-centered design).

**Fig 1:**
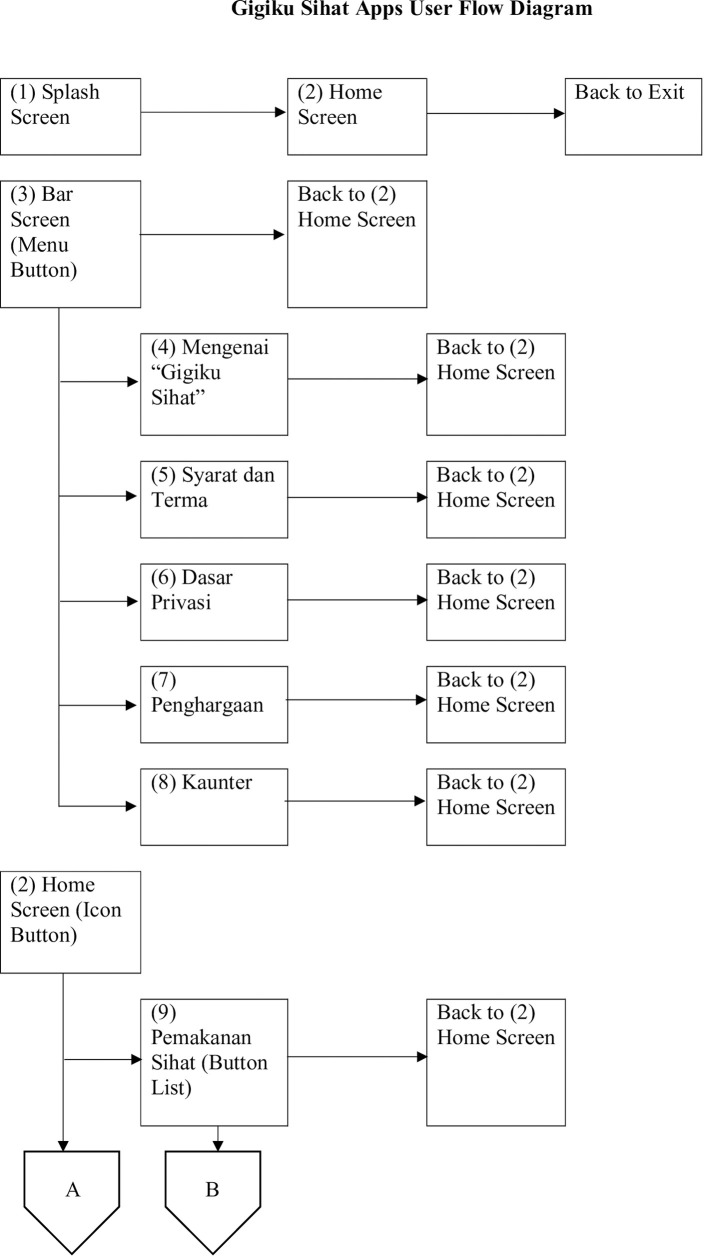
User flow diagram of *Gigiku Sihat*.

During the design phase, the researcher, designer and IT expert conducted face-to-face meetings and online consultations, which were scheduled from December 2018 to April 2019. The aim was to integrate the elements of successful UI, including clarity to prevent confusion, familiarity with functionality, navigational methods, and responsiveness including speed, aesthetics and interface continuity to enable users to understand patterns of usage. Resources used for the creation of *Gigiku Sihat* included Microsoft Words, Adobe Illustrator and Adobe XD. The process concerned was as follows:

*2*.*1*.*2*.*1 User flow diagram*. When the requirement was finalized, the research team with the advice of the IT expert created a *Gigiku Sihat* user flow diagram to chart and simulate the flow of the mobile application. The user flow diagram was drawn using Microsoft Words. The excerpt of the diagram is shown in Fig 1.

**Fig 2:**
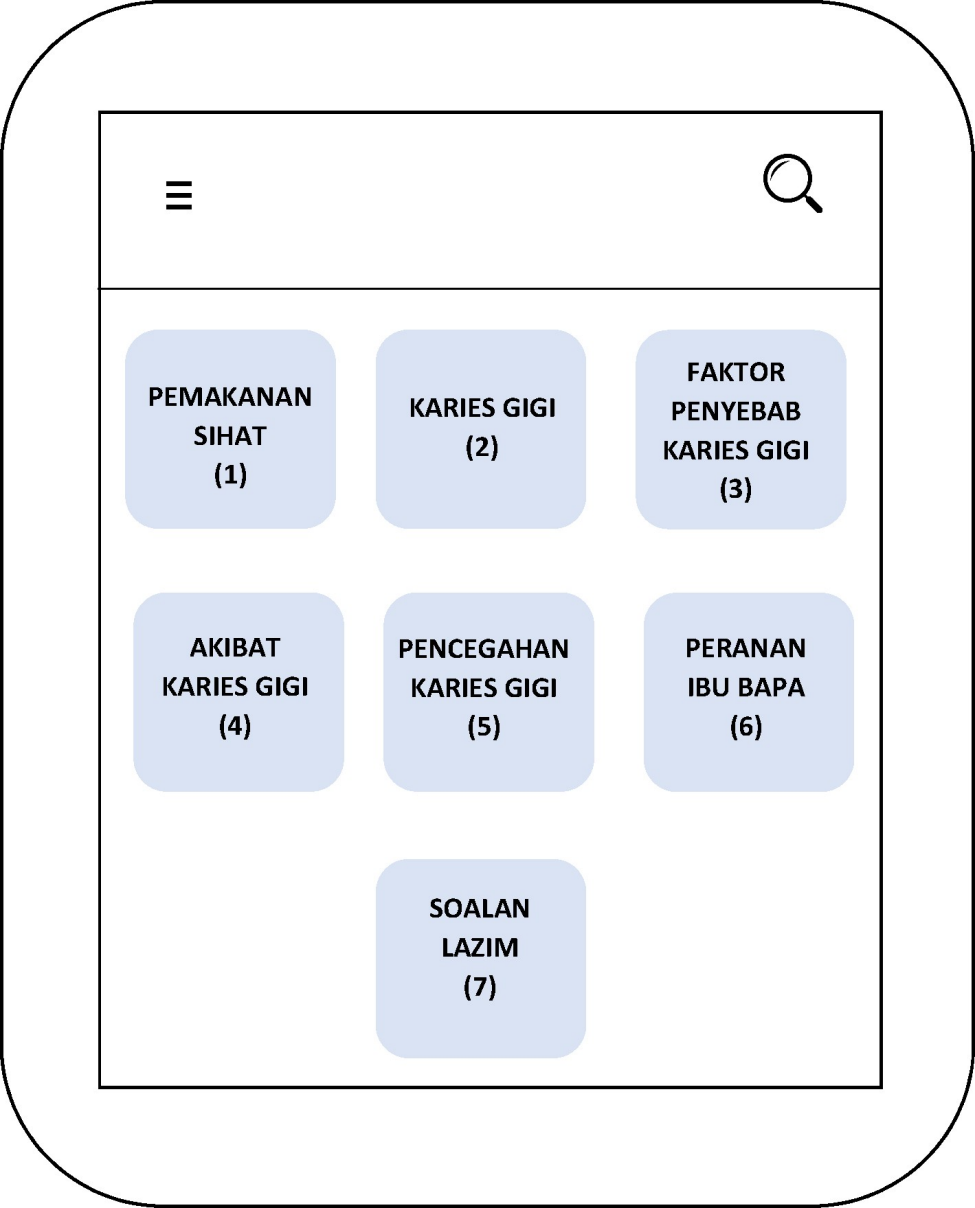
Microsoft words computer printed wireframe.

**Fig 3:**
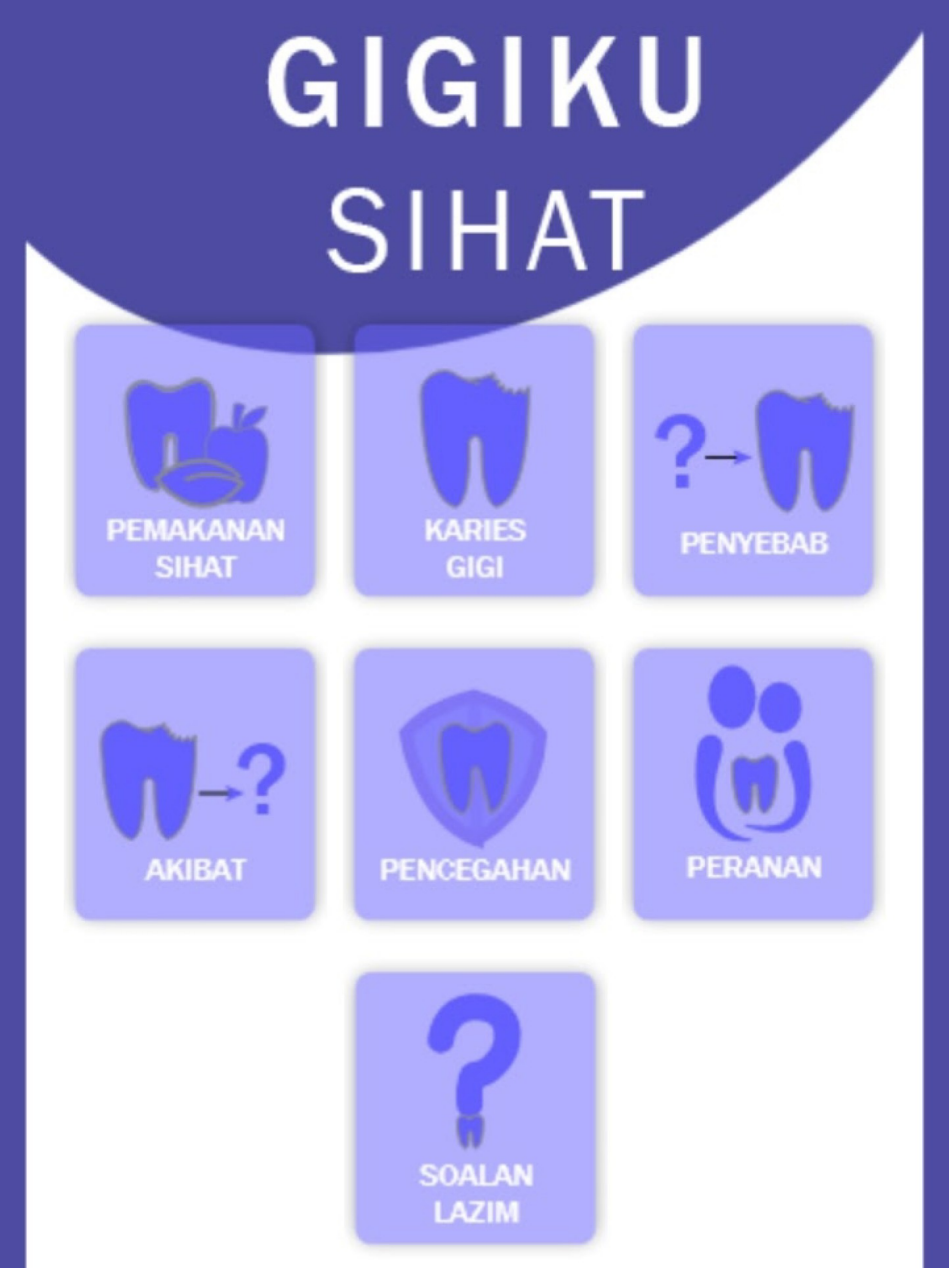
Adobe XD final design wireframe. Retrieved from https://xd.adobe.com/view/c2db4446-ddd7-4a6b-6b51-e4e6a4039098-b4ea/screen/e6040566-90e1-4a1f-a14b-42f1fd1e03de/.

*2*.*1*.*2*.*2 Wireframe*. The design implementation comprised of a low-fidelity wireframe and a high-fidelity wireframe. Wireframe, which was a condensed graphic depiction of the layout for the mobile application UI was developed in the early design phase. The research team created a low-fidelity wireframe utilizing Microsoft Words as a simple sketch for the *Gigiku Sihat* UI design. Subsequently, the high-fidelity wireframe (clickable mock-up) to simulate the navigation between the screens was developed by the researcher and design team using Adobe XD. Design evolutions are shown in Figs 2 and 3.

**Fig 4:**
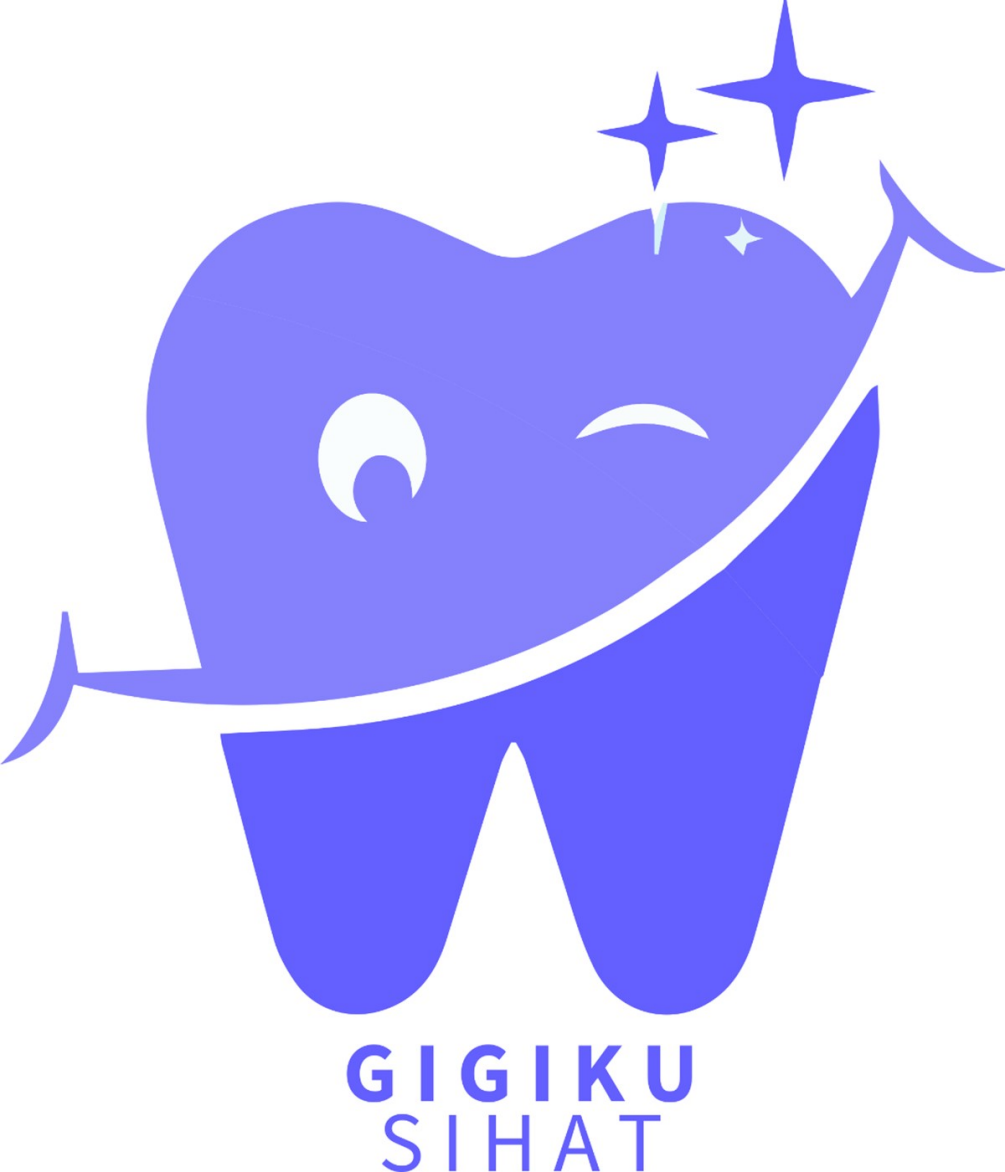
*Gigiku Sihat* logo.

*2*.*1*.*2*.*3 Gigiku Sihat logo*. As the researcher team planned to submit for copyright application of the mobile application, the logo for *Gigiku Sihat* was also designed in this phase using Adobe Illustrator. A minimal concept was chosen, in which two tone theme and Source Sans Variable typeface were used. The finalised logo is shown in Fig 4.

#### 2.1.3 Implementation

After the design phase was finished, we were able to build the *Gigiku Sihat* mobile application in the implementation phase. The mobile application prototype was developed for Android platform starting from April 2019. The program was coded at this phase. The coding of the mobile application was carried out using Android Studio by a mobile application developer with the advice of an IT expert. In order to meet the specifications set by the research team, *Gigiku Sihat* was built in Java programming language, and its application programming interfaces (APIs) were supported by Google. The mobile application database was developed into the Android-SQLite Database. The software programs for the Android operating system come in a format named the Android Application Package (APK) file. The finalized APK file was then used in the next phase of testing. The link for download was as follows: https://drive.google.com/u/0/uc?id=1V38QDSksRTwiVpJ9KtVwtwl0uHiipiws&export=download

**Fig 5:**
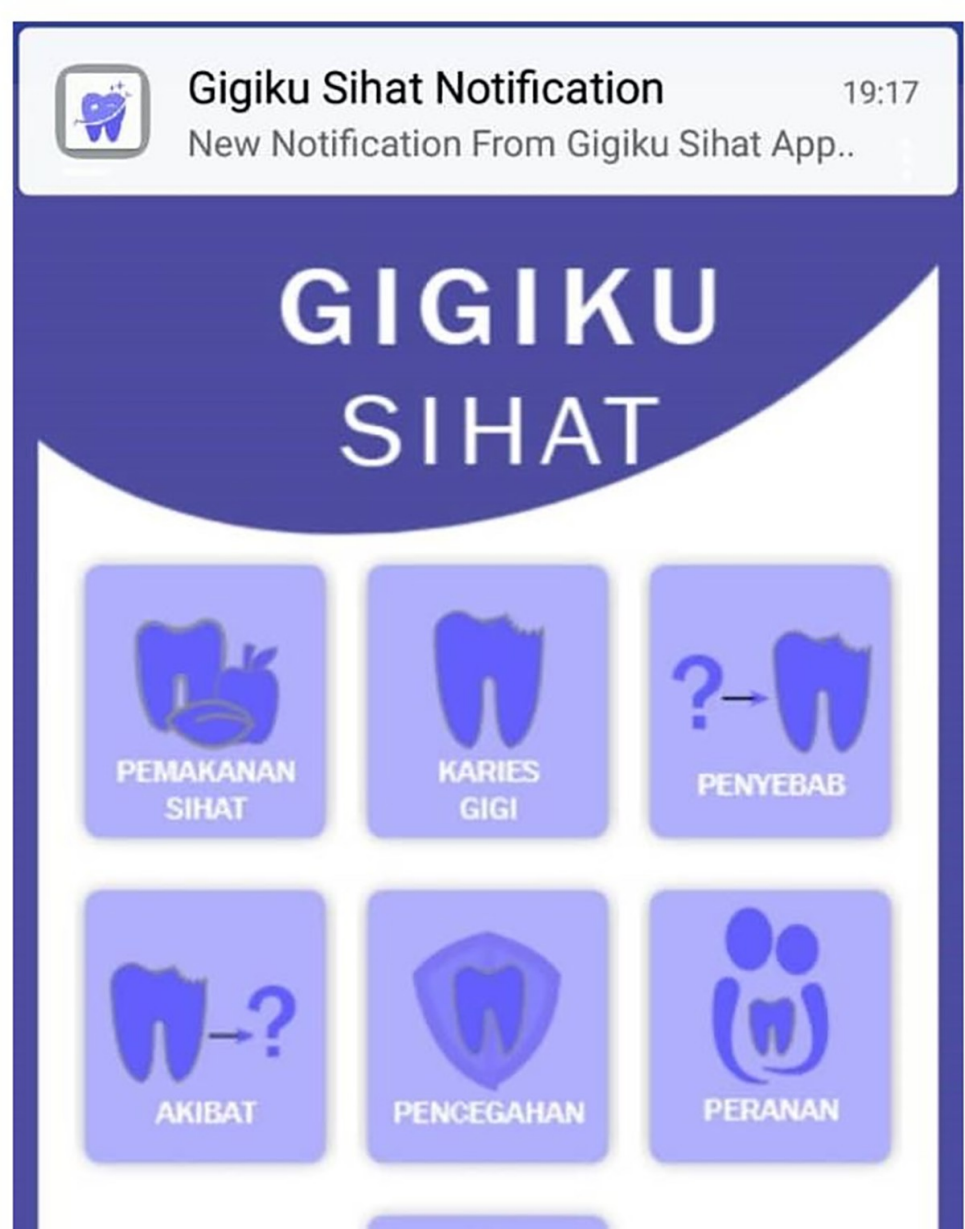
Status bar.

**Fig 6:**
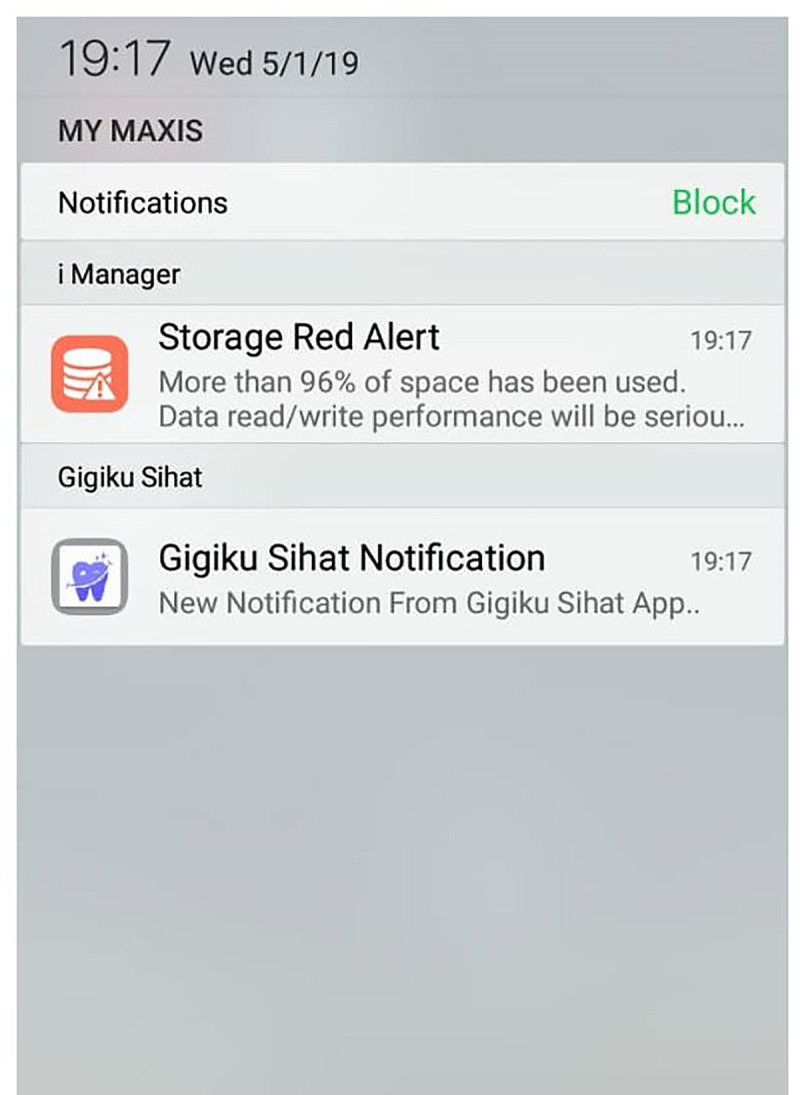
Notification drawer.

**Fig 7:**
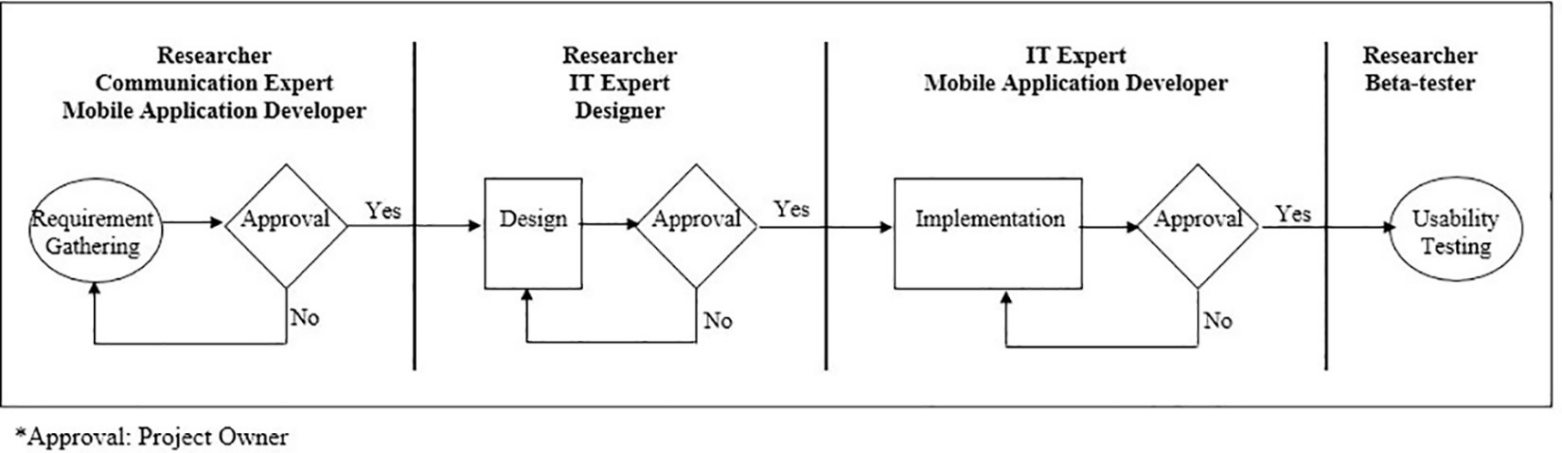
Development and usability testing responsibilities and process flow diagram.

Status bar and notification drawer were also included for the reminder and notification messages. When notification was issued, it first appeared as an icon in the status bar (Fig 5). Users could swipe down on the status bar to open the notification drawer, where they could view more details and take actions with the reminders and notifications (Fig 6). The overall development process flow is summarised in Fig 7.

### 2.2 Usability testing design

Mobile usability testing was carried out following conclusion of the implementation phase. Mobile application usability is defined based on the International Standards Organization (ISO) concept of usability as the degree to which a mobile application may be used by specified users to accomplish specified objectives with efficiency, satisfaction and effectiveness in a specified context of usage [61].

#### 2.2.1 Study population and design

A cross-sectional study was undertaken to determine the usability of the mobile application. The sample population included parents of 4 to 6-year-old children attending Dental Clinic at Hospital Universiti Sains Malaysia, Kubang Kerian. The inclusion criteria were parents of 4 to 6-year-old children and also owned a form of portable Android operating system, such as smartphones or tablets.

#### 2.2.2 Sample size determination

Ten beta testers were considered adequate to determine the usability of the mobile application. Selection of the number of beta testers was based on research results that suggested that ten participants in the usability test would recognise 94.7% of all usability concerns [62].

#### 2.2.3 Sampling method

The study sample of ten beta testers (parents of 4 to 6-year-old children) was obtained through convenience sampling. Apart from the confidentiality concern of the newly developed mobile application for a future trial, it was notable that the convenience sampling used for the study was the most commonly employed sampling methodology for usability testing [63, 64]. Recruitment of sample was based in Dental Clinic, Hospital Universiti Sains Malaysia, Kubang Kerian. Prospective beta-testers were approached face-to-face.

#### 2.2.4 Research tools

*Skala Kebolehgunaan Aplikasi Mudah Alih (SKAMA)*, the Malay language variant of the System Usability Scale (SUS) questionnaire was used in this analysis to test the usability for mobile application [65]. In practice, the use of well validated questionnaires designed for general software system such as SUS was also opted for questionnaire-based mobile app usability studies [66]. In addition, SUS was selected as it was already widely used and found to be remarkably robust on various studies, thus facilitating comparison.

The SKAMA questionnaire was readily translated, cross-culturally adapted and validated (CVI: 0.91; FVI: 0.94; Cronbach Alpha: 0.85) [65]. SKAMA has been used to assess the usability of mHealth application for community education [67]. It consisted of a 10-item survey rated from 1 (strongly disagree) to 5 (strongly agree) on a 5-point Likert scale, providing an overall subjective assessment of a system. The usability measurement included in SUS covered the effectiveness, efficiency and user satisfaction. The questionnaire was arranged to alternate positive and negative statements in order to eliminate a habitual bias on the part of the respondent.

We further classified a response as positive if the beta-testers chose score 5 or 4 for odd items (positive statements) and score 1 or 2 for even items (negative statements); neutral if beta-testers chose score 3; and negative if beta-testers chose score 1 or 2 for odd items and score 5 or 4 for even items. The contribution of the score to the even items was 5 minus the scale position and the contribution to the odd items was the scale position minus 1. In this study, the SKAMA score will be referred as “SUS score” to facilitate score comparison with other studies that use the same scale. The total SUS score was calculated out of the total number of item scores multiplied by 2.5 and it would vary from 0 to 100. It is understood that a score of 68 and above for a device or product reflects good usability [65].

#### 2.2.5 Data collection

When prospective beta-testers were approached, a clarification was provided by the researcher on the study and the procedures involved. Ten eligible and willing beta-testers were presented with the consent form to be completed. After the consent was obtained, sociodemographic information was acquired and *Gigiku Sihat* mobile application prototype was installed on their mobile devices by downloading the APK file using the QR code. Beta-testers were required to perform a series of tasks laid out in the usability test task sheet to ensure that all functionalities of the mobile application were used. A total of 10 tasks were given including starting the mobile application, navigating to the bar screen, navigating to the topic, navigating to the subtopic information, expanding the illustration or photograph, playing the video, browsing the frequently asked questions (FAQ), exiting the mobile application, opening the notification, and opening the reminder. The session was conducted in a room at the Dental Clinic, Universiti Sains Malaysia.

Beta-testers completed the series of tasks on their own, with the researcher acting as non-interactive observer for confirmation. Upon completion of the tasks, the beta-testers were required to complete a paper-based, self-administered questionnaire (*Skala Kebolehgunaan Aplikasi Mudah Alih-* SKAMA). The completed questionnaires were collected instantly. Subsequently, *Gigiku Sihat* was uninstalled from the beta-testers’ mobile devices utilizing the respective ’uninstall’ feature. The APK file ’Gigiku Sihat.APK’ was then removed using the search feature in the file manager folder. The questionnaires obtained were then prepared for data entry and analysis.

#### 2.2.6 Statistical analysis

The data were entered and evaluated using IBM SPSS Statistics Version 24. The data were summarized as frequency and percentage for categorical data, as well as mean and standard deviation (SD) for numerical data.

#### 2.2.7 Ethical statement

Ethical permission for this research was received from the Human Research Ethics Committee of Universiti Sains Malaysia (USM/JEPeM/19060364). This study was also registered in the National Medical Research Register of the Ministry of Health Malaysia (NMRR-20-2825-57812) and was given approval from the Oral Health Program of the Ministry of Health Malaysia (KKM-600-56/7/2 Jld.7 (37)).

## 3. Results

Usability testing of *Gigiku Sihat* was performed in August 2019. All of the participants were able to complete a total of ten tasks given within 5 minutes. The response rate for SKAMA questionnaire was 100%. The results were as follows:

### 3.1 Sociodemographic profile

All of the respondents were female. The majority of the respondents were above the age of 35 (70.0%), with a mean age of 37.1 (SD 3.45) years. Only one respondent completed post-secondary education, while others completed secondary (20.0%) and higher education (70.0%). The monthly household income for majority of the respondents (70.0%) was MYR3,000 and above. All respondents had experience using mobile applications, and majority (90.0%) had used mobile application at least once daily. Table 4 shows the sociodemographic characteristic of the beta-testers in this study.

**Table 4:** Sociodemographic characteristics of beta-testers (n = 10).

Variables	Frequency
	n (%)
**Age (years)**	37.1 (3.45)^a^
**Highest Education Level**	
SPM or equivalent	2 (20.0)
STPM/ Certificate or equivalent	1(10.0)
Diploma	5 (50.0)
Bachelor degree and above	2 (20.0)
**Monthly Household Income (MYR)**	
1,000–1,999	1 (10.0)
2,000–2,999	2 (20.0)
3,000–3,999	1 (10.0)
4,000–4,999	5 (50.0)
> 5,000	1 (10.0)
**Sex**	
Female	10 (100.0)
**Use of mobile application**	
Yes	10 (100.0)
**Frequency of use**	
At least once daily	9 (90.0)
At least once a week but not daily	1 (10.0)

^a^Mean (SD).

### 3.2 Mobile application usability

For all questions, the proportion of positive responses was higher than that of negative and neutral responses. The question with the most favourable answer (100.0% positive) was whether the mobile application was easy to use, whether the various functions were well integrated and whether they were able to learn how to use the mobile application quickly. The question with the least favourable responses was about the need to learn a lot of things before launching the mobile application (30.0% negative). It was also noted that the highest mean score was 3.4 (SD 0.52) for question five on the well integration of various functions. Meanwhile, the lowest mean score obtained was 2.5 (SD 1.27) for question ten on the need to learn a lot of things before launching the mobile application. The value of the SUS score for *Gigiku Sihat* was distributed between 55.0 and 100.0, with two respondents scoring below the minimum score. *Gigiku Sihat* mean (SD) usability score was 77.0 (14.18), higher than the minimum score for usable system which was 68 (Table 5). This score indicated good usability of *Gigiku Sihat* as a mobile application, which was also a usable tool for oral health education for parents of pre-school children.

**Table 5:** Mobile application usability (n = 10).

Variables	Positive responses	Neutral response	Negative responses	Score Mean (SD)
	n (%)	n (%)	n (%)	
**SKAMA Questions**				
Q1 –Like to use mobile application frequently	8 (80.0)	2 (20.0)	-	3.0 (0.67)
Q2 –Mobile application unnecessarily complex	8 (80.0)	2 (20.0)	-	3.3 (0.82)
Q3 –Mobile application easy to use	10 (100.0)	-	-	3.3 (0.48)
Q4 –Need assistance to use mobile application	8 (80.0)	-	2 (20.0)	3.0 (1.16)
Q5 –Various functions well integrated	10 (100.0)	-	-	3.4 (0.52)
Q6 –Too much inconsistency	7 (70.0)	1 (10.0)	2 (20.0)	2.8 (1.14)
Q7 –Learn to use mobile application very quickly	10 (100.0)	-	-	3.2 (0.42)
Q8 –Mobile application very cumbersome / awkward to use	8 (80.0)	2 (20.0)	-	3.0 (0.67)
Q9 –Confident using mobile application	9 (90.0)	1 (10.0)	-	3.3 (0.68)
Q10 –Needed to learn a lot of things before launching the mobile application	5 (50.0)	2 (20.0)	3 (30.0)	2.5 (1.27)
**Total responses**	83 (83.0)	10 (10.0)	7 (7.0)	
**Summation of Score**				30.8 (5.67)
**SUS Score**				(14.18)

## 4. Discussion

Usability testing decides the performance of these design decisions. In terms of *Gigiku Sihat* usability testing, this study had found that all of the respondents highly regarded that the mobile application was easy to use. According to Venkatesh et al., perceiving a mobile application as easy to use indicates the users’ intention to use it although they have no or little familiarity with the mobile application [68]. All respondents also unanimously agreed that various functions of *Gigiku Sihat* were well integrated. In this modern era, a single mobile application was expected to perform various functions in order to expedite access to information. In addition, shorter duration was needed by accessing various functions in a mobile application compared to the need to access multiple mobile applications to perform each single function.

Our findings that all respondents agreed that they were able to quickly learn how to use *Gigiku Sihat* validated the design process where the elements of successful UI were integrated. The possible explanation on the ability of the respondents to quickly learn how to use the mobile application might be due to the familiarity and consistency of *Gigiku Sihat* design that allowed the respondents to use its features intuitively. On the other hand, the most notable usability issue with *Gigiku Sihat* was the need to learn a lot of things before launching the mobile application as perceived by over a quarter of the beta-testers. This might be due the nature of this study where the mobile application was not available at a digital distribution platform such as the Google Play Store. The respondents had to download the APK file using the QR code in order to install *Gigiku Sihat* in their mobile devices. As this study is a part of a larger trial on the effectiveness of *Gigiku Sihat*, the results of this evaluation have important implications. To address the issue on learnability before using the mobile application, the researcher could assists the participants by providing guidance and technical support for the mobile application installation.

Overall, the beta testers feedback was positive with respect to the usability of the mobile application. The study found that the value of the SUS score for *Gigiku Sihat* was distributed between 55.0 and 100.0. We found that we could not easily explain why people assigned a poor or even high score without qualitative measures. Thus, while utilizing the SUS on its own would offer a basis of objective evidence to determine the usability of the mobile applications for researchers, open-ended questions may be applied at the end of the SUS if there is a need to further define unique problems with the mobile applications. The mean SUS score in this study, which was 77.0 was similar with the mean SUS score reported by Kortum and Sorber [69] among the top 10 mobile applications rated by 3,575 users. Sauro [70] clarified that seasoned users continued to have higher SUS scores relative to a first-time consumer. Therefore, it is possible that the level of overall SUS score in this study could be higher if the respondents were given more time and exposures to explore the mobile application. Further evaluation of different methodologies for the usability testing could be included, including those who have utilized the mobile application for some time.

In comparison to other prior studies on diet and oral health mobile application development and usability testing, this study contributes to the body of knowledge of the said domain with regards to linking diet and oral health for the content creation. This was reinforced by a study by Tiffany et al. [71] in which most mobile applications did not mention diets that were one of the main risk factors for oral diseases. Few documentations of development and usability studies of educational oral health mobile applications were available, hindering comparisons. Recently, Campos et al. [72] documented the development and usability testing of mobile application. However, the mobile application was directedly focused on pre-schoolers and featured gamification, and the usability testing was conducted with satisfaction interview and observation suitable to their target group. In fact, the most recent systematic search and quality appraisal study concluded that there was no high-quality mobile applications aimed at parents to highlight the particular dental caries preventive activities for children aged 6 years and below [73].

Patient education in disease management and prevention by smartphones have been shown to be efficient and easy [74, 75]. Fundamentally, the development and usability testing process was critical to optimize the mobile applications as an oral health education tool. In this study, the System Development Life Cycle (SDLC) principles offered good criteria for implementers to adopt. *Gigiku Sihat* was created by a multidisciplinary teams as it was the best practice methodology suggested by several researchers [76, 77]. Furthermore, collaborating with mobile application development experts early in the project would help ensure that the mobile applications were well designed and implemented [78].

The rational model and the health belief model have been integrated into the *Gigiku Sihat* mobile application to further improve the acceptance of healthy oral health behaviours. Notably, the health belief model was the most widely quoted theory as explained in a systematic review by Cho et al. [79] on the theories applicable to mHealth intervention for behavioural improvement by low- and middle-income countries. In particular, cues to action concept by employing push notifications were used in this paper as one of the possible methods for behaviour improvement. Compared to other notification types such as e-mail, push notifications are instant and easy to act where swiping the notification brings consumers straight to the mobile application. Notifications will also stay in the list until they are withdrawn or actuated, which ensuring that they may actually act as prompts for further actions.

Increased awareness of the usability of the mobile applications will help to provide valuable information for their continued growth [69]. However, there are several limitations to consider in this study. The participants in the usability testing were aged between 32 and 43 years, meaning that younger parents were not represented in this sample. This was due to the convenience sampling method used. Although we recruited only 10 participants for the testing phase, this amount has been previously shown to be effective in identifying most usability issues. As such and because of the small number of users rating the mobile application, these results should be interpreted with caution. It also should be highlighted that despite the value of this robust development process, SDLC was very time-consuming because the approval was needed for each phase, and progress to another phase was halted if approval was not consented in the previous phase.

Development of this mobile application focusing on diet and oral health serves as a new source of oral health education for parents in the prevention of early childhood caries. This study also provides a necessary foundation to develop future improved diet and oral health mobile application. As caries prevalence in Malaysian pre-school children is still high, this mobile application can be used as a medium for oral health promotion under the Ministry of Health Malaysia. As a way forward, the mobile application will be used in a larger trial to determine the effectiveness of *Gigiku Sihat*.

## 5. Conclusions

*Gigiku Sihat* was successfully developed after undergoing phases of requirement gathering, design, implementation and usability testing. It is informative with four key messages in oral health tailored for local parents or guardians of pre-school children. It is also interactive with features such as graphics, videos and push notifications. Integrated content and design were developed by a multidisciplinary team. The highest mean score was achieved for good integration of various mobile application functions. To address the issue on learnability before using the mobile application, the researcher could assists the participants by providing guidance and technical support for the mobile application installation. Based on the results, it can be concluded that *Gigiku Sihat* has a good usability as an oral health education for parents or guardians of pre-school children.

## Supporting information

S1 Table List of notification and reminder messages. (PDF)
