# Supplementary material for: Development and usability testing of mobile application on diet and oral health
Source: PLoS One. 2021 Sep 8;16(9):e0257035. doi: 10.1371/journal.pone.0257035 (PMC8425540; doi:10.1371/journal.pone.0257035)
Supplement: S1 Table — (PDF) [file pone.0257035.s001.pdf]

S1 Table. List of Notification and Reminder Messages

No.	Notification Messages
1.	Gula yang terdapat dalam susu, jus buah-buahan atau minuman bergula yang lain akan bertindak balas dengan bakteria dalam mulut boleh menghasilkan asid yang menyebabkan kerosakan gigi.
2.	Tanda-tanda awal kejadian karies yang dapat dilihat di dalam mulut adalah tompok atau garisan putih (*white spot lesions*) pada permukaan gigi yang tersembunyi di bawah plak.
3.	Apabila kanak-kanak sudah boleh mula meludah, penggunaan ubat gigi berfluorida boleh digunakan.
4.	Amalkan pemakanan yang seimbang, lebihkan buah-buahan dan sayur-sayuran segar dalam diet harian anak anda.
5.	Kenyataan yang mengatakan epal adalah pembersih gigi adalah salah kerana dengan memakan epal, tidak boleh membersih plak yang ada di gigi.
6.	Kurangkan pengambilan gula dalam semua makanan dan minuman. Minuman berperisa buah-buahan (contoh: laici, anggur, oren dan lain-lain) adalah tidak selamat untuk gigi anak anda.
7.	Elakkan pengambilan makanan bergula di antara waktu makan utama. Sekiranya tidak dapat dielakkan, pilih makanan kariostatik yang tidak merbahaya seperti buah-buahan segar, kekacang, susu, dan keju.
8.	Elakkan mengambil minuman bergas/berkarbonat dan isotonik terlalu kerap kerana ia mempunyai kandungan gula yang tinggi.
9.	Berus gigi anak anda sekurang-kurangnya 2 kali sehari. Galakkan juga anak-anak anda berkumur selepas makan untuk mengeluarkan sisa makanan/minuman bergula.
10.	Elakkan menambah gula ke dalam susu formula anak anda. Elakkan memberi susu formula yang mengandungi gula tambahan kepada anak anda.
11.	Jangan biarkan bayi atau kanak-kanak tidur dengan botol yang mengandungi susu, jus buah-buahan atau minuman bergula lain kerana ia boleh merosakkan gigi.
12.	Penggunaan botol susu sebaiknya dihentikan setelah anak berumur 1 tahun.
13.	Hadkan makanan/minuman bergula pada waktu makan utama iaitu sarapan, makan tengahari dan malam sahaja.
14.	Elakkan memberi gula-gula, manisan, biskut dan ais krim sebagai hadiah atau ganjaran kepada kanak-kanak.

No.	Notification Messages
15.	Lawatan pertama ke klinik untuk pemeriksaan perlu dilakukan dalam tempoh 6 bulan selepas gigi pertama tumbuh atau sebelum umur bayi mencecah 1 tahun.
16.	Kebanyakan ubat dalam bentuk cecair dan vitamin kanak-kanak mempunyai kandungan gula yang tinggi. Kumur setelah mengambil ubat-ubatan tersebut.
17.	Pemeriksaan gigi perlu dilakukan secara berkala sekurang-kurangnya sekali setahun. Pemeriksaan atau lawatan yang lebih kerap akan disyorkan jika perlu.
18.	Beri makanan yang mencukupi mengikut piramid makanan bagi keperluan tenaga anak anda.
19.	Semak kandungan gula pada senarai ramuan sesuatu produk makanan. Sekiranya gula tersenarai di bahagian awal senarai ramuan, ini menunjukkan gula merupakan komponen utama makanan tersebut.
20.	Beri pelbagai jenis makanan bagi memastikan keperluan nutrien kanak-kanak dipenuhi.
21.	Jangan beri minuman manis dalam botol susu.
22.	Satu tin minuman bergas (12 oz) mengandungi 10 sudu teh gula dan ia boleh merosakkan gigi anak anda
23.	Pilih makanan yang rendah gula. Makanan jenis ini biasanya dilabel sebagai kurang gula (*less sugar*) atau bebas gula (*sugar-free*).
24.	Galakkan penggunaan cawan seawal usia 6 bulan dan gunakan cawan sepenuhnya untuk menggantikan botol susu apabila anak telah mencapai usia 12 bulan.
25.	Ubat gigi berfluorida baik untuk anak anda kerana ia boleh mencegah gigi berlubang.
26.	Selia dan bantu anak anda memberus gigi sehingga mereka berusia enam hingga lapan tahun.
27.	Pilih berus gigi yang sesuai, bersaiz kecil dan lembut dan mempunyai pemegang yang selesa untuk anak anda.
28.	Flourida melindungi gigi dengan membuat enamel lebih berdaya tahan daripada serangan asid.
29.	Pantau kesihatan gigi anak anda secara berkala.
30.	Pilihlah jenis makanan dan kuantiti yang betul mengikut saranan pinggan Suku-Suku-Separuh dengan penyediaan yang menarik untuk menjamin pertumbuhan yang optimum untuk anak anda.

No.	**Notification Messages**
31.	Kurangkan kekerapan pengambilan gula. Sekiranya anak anda mempunyai gula-gula yang banyak, adalah baik untuk dimakan semuanya sekaligus.
32.	Karies di peringkat awal kanak-kanak dapat dicegah melalui penjagaan kebersihan mulut dan mengelakkan makanan bergula.
33.	Elakkan mengambil makanan yang manis dan melekit.
34.	Bersihkan gigi anak anda sebaik sahaja gigi tumbuh.
35.	Elakkan mengambil makanan manis secara berterusan iaitu dalam jangkamasa kurang dari 20 minit.
36.	Berilah anak anda diet harian yang seimbang dan berkhasiat iaitu dengan pengambilan makanan dengan mengikuti saranan yang terdapat dalam piramid makanan.
37.	Gunakan hanya secalit ubat gigi sahaja untuk kanak-kanak berumur di bawah 3 tahun.
38.	Pastikan anak anda tidak menelan ubat gigi semasa memberus gigi.
39.	Elakkan mencelup puting susu dengan gula atau manisan.
40.	Gunakan ubat gigi sebesar kacang pis (5mm) sahaja untuk kanak-kanak yang berumur 3 tahun ke atas.
41.	Gunakan susu segar atau susu rendah lemak bagi menggantikan susu pekat manis.
42.	Sentiasa memerhatikan anak anda semasa memberi susu di dalam botol dengan mengikut masa yang telah ditetapkan
43.	Gigi kanak-kanak perlu dibersihkan sekurang-kurangnya 2 kali sehari, iaitu pada waktu pagi dan pada waktu malam sebelum tidur.
44.	Keluarkan botol susu dari mulut sebaik sahaja habis menyusu botol.
45.	Sebelum tumbuhnya gigi susu, bersihkan gusi anak-anak menggunakan tuala atau kain bersih yang dibasahkan dan dibalut pada jari.
**No.**	**Reminder Messages**
1.	Anda belum log masuk ke aplikasi “Gigiku Sihat” untuk minggu ini. Anda ingin melakukannya sekarang? Klik di sini
2.	Salam Sejahtera! Dah lama tak jumpa tuan/puan di aplikasi Gigiku Sihat. Jom log masuk di sini.
